# Hæmatology—VIII

**Published:** 1910-09-24

**Authors:** 


### PATHOLOGY.

### HÆMATOLOGY—VIII.

### Anæmia and Polycythæmia.

Anæmia is usually divided up into primary and secondary, the former title being employed for two diseases (chlorosis and pernicious anæmia) the etiology of which so far is speculative, the latter for those where there is a definite cause demonstrable during life or at autopsy. If pernicious anæmia is simply a result of oral sepsis, as Hunter believes, then it must take its place in the secondary group, and only chlorosis will be left in the primary. In anæmia many abnormally shaped or sized red corpuscles are met with, and different names have been given to these, names which are puzzling or even unintelligible to the beginner, so they may be explained first. The normal red cell is called an *erythrocyte* (*e.g.* a non-nucleated bi-concave circular disc measuring 7.5 $\mu$ in diameter). If the size falls below this, then the cell is termed a *microcyte*; if it rises above it, a *megalocyte*; and if the shape is irregular and no longer circular, a *poikilocyte*. Nucleated red cells are termed blasts; so, using the same nomenclature, we have the *eyrthroblast* (normoblast), the normal-sized red with a nucleus; the *microblast*, the small red with a nucleus; and the *megaloblast*, the large red with a nucleus. *Polychromatophilia* is a term used to indicate that the red cell has taken up different stains (basic and acid parts); normally it should only take up the acid part, the eosin, and stain pink, whereas in the former it stains purple. *Basophilia* is another name; it indicates a granular (basophile) degeneration seen in the red cells in various anæmias, which gives the cell the appearance of being stippled with fine little blue points. Various red spots or dots are also seen in malarial bloods (benign tertian and drepanidium of the frog) in the cells containing parasites; these are termed Schüffner's dots.

### Primary Anæmias.

*Chlorosis.*—This is a disease found in young females, the name being derived from the peculiar yellow-green tinge of the skin which accompanies it The usual symptoms of anæmia—breathlessness on exertion, giddiness, palpitation, fainting, &c.—are all present. The main changes, and especially those on which the diagnosis is to be founded, occur, however, in the blood. These essentially are a great diminution of the hæmoglobin as compared with the red corpuscles, diminution of the leucocytes, a fair degree of poikilocytosis, a nucleated red now and again, and a low specific gravity. Actual counts are as follows: (1) R. 4,000,000, W. 3,000, Hb. 25 per cent. (*e.g.* 80 per cent. of reds, only 25 per cent. of hæmoglobin); (2) R. 3,400,000, W. 4,000, Hb. 30 per cent. (68 per cent. reds, 30 per cent. hæmoglobin), giving a negative colour index. Under suitable treatment—rest in bed, iron and arsenic, keeping the bowels open, etc.—the disease can be cured. In those who get married it is said to disappear of its own accord.

*Pernicious Anæmia.*—In contradistinction to chlorosis, pernicious anæmia is not limited to one sex, males and females being about equally attacked, and also old people as well as young. The colour of the skin is generally of a distinct lemon tint, though in other cases, especially in very advanced ones, it may have a dead white or waxy appearance. Insidious in its onset, the disease soon comes to show the ordinary clinical signs of anæmia, and in its later stages debility, œdema, and hæmorrhages are usually met with. The main changes in the blood are the excessive poikilocytosis, a normal or increased hæmoglobin per corpuscle (a + colour index), great diminution of leucocytes, a relative increase of the lymphocytes, nucleated reds common, and sometimes a few myelocytes present. A typical count would be as follows: R. 2,000,000 (40 per cent.), W. 2,200, Hb. 60 per cent., colour index + 1.5; lymphocytes in the differential counts over 40 per cent.; microcytes, megalocytes, polychromatophilic cells, and nucleated reds—normoblasts or megaloblasts—all present. The prognosis of the disease is bad. Atoxyl, arsenic, bone marrow, and some other drugs temporarily produce improvement, this, however, usually being followed by relapses and so on until the fatal termination. Some cases run an acute course, others a chronic course even of years.

### Secondary Anæmias.

The main points of a secondary anæmia are the equal diminution of the hæmoglobin to the reds, giving a colour index of somewhere about 1; special diagnostic features, such as leucocytosis, normal leucocyte counts or leucopenia; differential increases in large mononuclear cells, eosinophiles, and so on may be found. The presence or absence of any of these, taken with the clinical history of the case, may be of the greatest value in coming to a correct diagnosis. Any debilitating or wasting disease will quickly produce anæmia, be they due to bacteria of different kinds, protozoa, or other poisons. Of such the number is legion—cancer, Bright's disease, sepsis (liver abscess, empyema, etc.), late stages of typhoid and Malta fever, hæmorrhages (continued), syphilis, malaria, kala azar, bothriocephalus, ankylostomiasis, bilharzia, lead, mercury, and arsenical poisoning, and so on.

*Ankylostomiasis Anæmia.*—The presence of a large number of ankylostomes in the intestine may give rise to a marked anæmia which may often be mistaken for real pernicious anæmia. The points to attend to are the fact that the patient has been abroad, a relative increase of the eosinophiles 18 to 20 per cent., and a colour index of about normal (the statement that a diminished colour index is present in this disease is wrong).

*Malarial Anæmia.*—In chronic cachectic cases this may be extreme, for example: reds 2,300,000 (46 per cent.), W. 3,000, Hb. 45 per cent., differentially large mononuclears 20 per cent. Parasites, if found, clinch the diagnosis, but if absent, through large doses of quinine, then such a count should still raise one's suspicions.

*Suppuration Anæmia.*—In this form there should be a leucocytosis. Example: R. 3,400,000, W. 22,000, Hb. 70 per cent.

Polycythæmia.

Osler some years ago brought this condition into prominence by describing three cases of the condition known as "Chronic Cyanotic Polycythæmia" (with enlarged spleen). Since that time other cases have been recorded showing that the disease is not so rare as was at first supposed. The following is a synopsis of its chief characteristics. Symptoms: fulness in the head, headache, vertigo, weakness, prostration, sometimes nausea and vomiting. The blood: increased viscidity, very dark colour, R.b.c. about 9,000,000 per c.mm., Hb. 120 to 150 per cent., leucocytes no change (sometimes increased 16,000 in one case), spleen enlarged. Though chronic cardiac disease with emphysema, primary tuberculosis of the spleen, and chronic poisoning with coal-tar products may produce a cyanosis and polycythæmia, yet these were all absent in Osler's and other cases, therefore one must look upon the disease as a distinct clinical entity.

